# Exosomal lncRNA SNHG12 promotes angiogenesis and breast cancer progression

**DOI:** 10.1007/s12282-024-01574-6

**Published:** 2024-06-04

**Authors:** Yan Chen, Yuxin Zhou, Jiafeng Chen, Jiahui Yang, Yijie Yuan, Weizhu Wu

**Affiliations:** 1 Department of Thyroid and Breast Surgery, The Affiliated Lihuili Hospital, Ningbo University, Ningbo, 315000 China; 2 East Branch of Lihuili Hospital, Ningbo Medical Center, No. 1111 Jiangnan Road, Meixu Street, Yinzhou District, Ningbo, Zhejiang China

**Keywords:** Breast cancer, Exosomes, lncRNA SNHG12, Angiogenesis, Progression

## Abstract

**Objective:**

Breast cancer is one of the most prevalent malignancies in women. Exosomes are important mediators of intercellular communication; however, their regulatory mechanisms in human umbilical vein endothelial cells (HUVECs) angiogenesis in breast cancer remain unknown.

**Methods:**

We isolated and characterized breast cancer cell-derived exosomes and investigated their functions. Exosomal sequencing and the TCGA database were used to screen long non-coding RNA (lncRNA). In vitro and in vivo experiments were performed to investigate the role of exosomal lncRNA in HUVEC angiogenesis and tumor growth. Molecular methods were used to demonstrate the molecular mechanism of lncRNA.

**Results:**

We demonstrated that breast cancer cell-derived exosomes promoted HUVEC proliferation, tube formation, and migration. Combining exosomal sequencing results with The Cancer Genome Atlas Breast Cancer database, we screened lncRNA small nucleolar RNA host gene 12 (SNHG12), which was highly expressed in breast cancer cells. SNHG12 was also upregulated in HUVECs co-cultured with exosome-overexpressed SNHG12. Moreover, overexpression of SNHG12 in exosomes increased HUVEC proliferation and migration, whereas deletion of SNHG12 in exosomes showed the opposite effects. In vivo experiments showed that SNHG12 knockdown in exosomes inhibited breast cancer tumor growth. Transcriptome sequencing identified MMP10 as the target gene of SNHG12. Functional experiments revealed that MMP10 overexpression promoted HUVEC angiogenesis. Mechanistically, SNHG12 blocked the interaction between PBRM1 and MMP10 by directly binding to PBRM1. Moreover, exosomal SNHG12 promoted HUVEC angiogenesis via PBRM1 and MMP10.

**Conclusions:**

In summary, our findings confirmed that exosomal SNHG12 promoted HUVEC angiogenesis via the PBRM1-MMP10 axis, leading to enhanced malignancy of breast cancer. Exosomal SNHG12 may be a novel therapeutic target for breast cancer.

**Supplementary Information:**

The online version contains supplementary material available at 10.1007/s12282-024-01574-6.

## Introduction

Breast cancer, the most common type of cancer, is one of the most serious malignancies threatening women’s health [1]. Breast cancer treatment has become more diverse as medicine has advanced, including traditional surgery, chemotherapy, radiotherapy, and comprehensive treatment [2]. Although these treatments can benefit the survival of most patients, many breast cancer patients die as a result of disease progression [3]. According to studies, 20–30% of breast cancer patients develop distant metastases after diagnosis [4], and tumor angiogenesis is one of the important factors leading to recurrence and metastasis of breast cancer [5].

Angiogenesis is a key process contributing to breast cancer development [6]. Tumor angiogenesis is a process in which the balance between pro- and antiangiogenic factors is disrupted [7]. Cancer cells can obtain nutrients required for growth and spread as a result of neovascularization, leading to tumor formation, progression, and metastasis [8]. As a result, inhibiting tumor angiogenesis is an important therapeutic target for cancer treatment because it reduces the oxygen and nutrient supply required for tumor growth [9]. However, the mechanism of angiogenesis in breast cancer is not fully elucidated yet.

Exosomes are nanoscale lipid inclusions with a diameter of 30–150 nm that transport proteins, DNA, miRNAs, and other non-coding RNAs to mediate intercellular communication [10, 11]. Exosomes are naturally found in bodily fluids, such as blood, saliva, urine, and breast milk [11], making them potential biomarkers for the early detection of a variety of diseases [12]. Exosomal lncRNA has been reported to participate in tumor development, including growth, metastasis, and angiogenesis [13]. For example, ovarian cancer cell-derived exosomal lncRNA ATB promoted ovarian cancer growth and angiogenesis via the miR-204-3p/TGFβR2 axis [14]. Exosomal lncRNA SNHG11 facilitated angiogenesis in pancreatic carcinoma by sponging miR-324-3p and targeting VEGFA [15]. Exosomal LINC00161 accelerated angiogenesis and metastasis in hepatocellular carcinoma by modulating the miR-590-3p/ROCK axis [16]. However, the role of breast cancer-derived exosomal lncRNAs in angiogenesis remains unknown.

In this study, we conducted in vitro and in vivo experiments to confirm the effect of exosomal lncRNAs on breast cancer angiogenesis and the specific regulatory mechanism, in order to develop a safe and effective therapeutic method for breast cancer.

## Materials and methods

### Data collection and bioinformatics analysis

Based on our previous sequencing results [17], differentially expressed lncRNAs in exosomes from breast cancer cells and normal breast epithelial cells were obtained using the threshold value (log_2_ (fold change, FC) > 1.25 for upregulated lncRNAs and *P* < 0.05). mRNAs with differential expression in breast cancer and normal tissues were identified using |log_2_ (FC)| > 1.25 and *P* < 0.05 in the TCGA database (https://cancergenome.nih.gov). DAVID was utilized to perform gene ontology (GO) and Kyoto Encyclopedia of Genes and Genomes (KEGG) analysis. Cytoscape version 3.6.1 was used to establish a network.

### Cell culture

The breast cancer cell line MDA-MB-468 was purchased from the Chinese Academy of Sciences (Shanghai, China). The breast cancer cell line MDA-MB-231, MCF-7 and normal breast epithelial cell MCF-10A were purchased from Procell (Wuhan, China). The human umbilical vein endothelial cells (HUVECs) were purchased from iCellbioscience (Shanghai, China). MDA-MB-231 cells, and MCF-7 cells were cultured in the Dulbecco’s Modified Eagle’s Medium (Corning, #10-013-CVR). MDA-MB-468 cells were cultured in the L-15 Medium (Solarbio, # LA9510). MCF-10A cells were cultured in the MCF-10A cell specific medium (Procell, #CM-0525). HUVECs were cultured in the ScienCell 1001 medium. All medium was added with 10% fetal bovine serum (Gibco) and 1% penicillin/streptomycin (Sangon). All cells were incubated at 37 °C with 5% CO_2_.

### Quantitative real-time PCR (qRT-PCR)

Total RNA was extracted from cells and exosomes using TRIzol reagent (Invitrogen, USA). cDNA was prepared through the reverse transcription reaction with the cDNA Synthesis Kit (Thermo). SYBR Green PCR Master Mix (Roche) was applied for qRT-PCR analysis. The lncRNA and mRNA expression were normalized to GAPDH. The primer sequences are listed in Table S1.

### Isolation and characterization of exosomes

MDA-MB-231 cells and MCF10A cells were cultured in medium without FBS. The culture supernatants were centrifuged at 300×*g* for 10 min, followed by centrifugation at 2000×*g* for 20 min to remove the cells. Cell debris was removed by centrifuging the supernatant at 10,000×*g* for 30 min. The exosomes were extracted by ultracentrifugation at 100,000×*g* for 1.5 h, and the precipitates were collected. Transmission electron microscopy (TEM) was used to observe exosome morphology and particle size. In brief, exosome was resuspended in 4% paraformaldehyde, and images were obtained using the JEOL 100 CX electron microscope (Tokyo, Japan). The protein concentration of exosomes was determined using a Modified BCA Protein Assay Kit (C503051, Sangon Biotech).

### Western blot

RIPA lysis buffer (Thermo Fisher) was used to extract proteins from MDA-MB-231 cells, MCF-10A cells, corresponding exosomes, and HUVECs. The samples were washed with 1× PBS and resuspended in SDS lysis buffer, and then centrifuged at 16,000×*g* for 30 min and the supernatant was collected. The remaining steps were carried out as reported previously [18]. The following antibodies were used: GAPDH (Proteintech, #60004-1-Lg, 1:2000); CD63, (Abcam, #ab216130, 1:1000); PBRM1 (Proteintech, #12563-1-AP, 1:1000); goat anti-rabbit IgG H&L(HRP) (Beyotime, #A0208, 1:1000); and goat anti-mouse IgG H&L(HRP) (Beyotime, #A0216, 1:1000).

### CCK-8 assay

A CCK-8 assay was utilized to evaluate the proliferation ability of HUVECs. Exosome-treated HUVECs were digested into a single-cell suspension (1 × 10^4^/mL). The cells were seeded into 6-well plates at a density of 10^3^ cells/well and cultured overnight at 37 °C. The cells were then added with 10 μL CCK-8 solution. After 2 h, the absorbance value was measured at 450 nm.

### Wound healing assay

A wound healing assay was utilized to evaluate the migration ability of HUVECs. HUVECs were seeded into 24-well tissue culture plates at a density of 8 × 10^4^ cells/well and cultured overnight. After the cells were scratched, they were washed with PBS to remove the detached cells. Subsequently, serum-free medium was added, followed by exosomes derived from MDA-MB-231 and MCF-10A for 24 h of incubation. At 0 and 24 h, the monolayer cells were photographed using a Nikon microscope.

### Tube formation assay

A tube formation assay was utilized to evaluate the tube formation ability of HUVECs. The cooled 24-well plates were filled with 200 μL of cooled Matrigel (10 mg/mL) and placed in a 37 °C incubator for 1 h. HUVECs were digested into cell suspensions, and 8 × 10^4^ cells were seeded on coagulated Matrigel gel, followed by the addition of exosomes for 24 h of incubation (37 °C, 5% CO_2_). The 24-well plate was taken out and photographed under a microscope.

### Cell transfection

The pcDNA3.1-CMV-SNHG12, pcDNA3.1-CMV-PBRM1 and pcDNA3.1-CMV-MMP10 plasmids were purchased from Hanbio Biotechnology. SiRNA-SNHG12 and siRNA-MMP10 were designed and synthesized by RiboBio (Guangzhou, China). Lipofectamine 2000 (Invitrogen) was used to transfect the plasmids into MDA-MB-231 cells or HUVECs, and DharmaFECT4 (Dharmacon, USA) was used to transfect the siRNAs.

### Transwell assay

A transwell assay was utilized to evaluate the migration ability of HUVECs. After removing the medium, cells were washed with an appropriate amount of PBS, and added 0.25% Trypsin–EDTA for digestion 3 min. After the cells shrank and became round, the digestion was terminated by adding culture medium. Thereafter, cells were centrifuged at 200×*g* for 3 min, and the supernatant was removed. Cells were resuspended with serum-free medium. Next, 700 μL of medium containing 10% FBS was added to the lower chamber, and 500 μL of cell suspension and 20 μg/mL exosomes were added to the upper chamber. The culture was continued in the CO_2_ incubator for 24 h. Subsequently, the upper chamber was added with 4% polyformaldehyde and fixed at room temperature for 30 min, followed by crystal violet staining. Finally, the cells were counted under a microscope (OLYMPUS, CKX53).

### Tumor formation in nude mice

All animal experiments were approved by the Animal Care Committee of Ningbo University. Female athymic BALB/c nude mice (5 weeks, 20 ± 2 g) were obtained from the Chinese Academy of Science. MDA-MB-231 cells were seeded subcutaneously on each flank of the nude mice. A tumor with a size of 100 mm^3^ was formed subcutaneously, and the collected exosomes of knockdown SNHG12 and the corresponding empty vector were injected into the tumor site once a week (*n* = 5). After 26 d, the mice were sacrificed, the tumor volume and weight were measured, and samples were collected to detect SNHG12 and microvessel density (MVD).

### Immunofluorescence analysis

Immunofluorescence analysis was performed as previously described [19]. Briefly, the tumor tissue was fixed, followed by dehydration, embedding, sectioning (4 μm), dehydration, antigen retrieval, and blocking. Immunofluorescence analysis was performed using anti-CD31 (Abcam, #ab222783), anti-CD34 (Abcam, #ab81289), and Cy3 conjugated Donkey anti-rabbit IgG (H + L) (Servicebio, #GB21403) antibodies. After DAPI staining, the images were observed under a fluorescence microscope (Nikon).

### RNA sequencing

The RNA of HUVECs transfected with the SNHG12-overexpressing vector or control vector was extracted for library construction using KAPA Stranded RNA-seq library Prep Kit (Illumina, USA). The Illumina NovaSeq 6000 Sequencing system (Illumina) was applied to pair-end sequence the library. The transcriptome sequencing experiment was completed by Mingma Technologies Co., Ltd. (Shanghai, China). Adapter sequences were filtered to obtain clean reads. The sequencing results were then aligned to a reference genome GRCh38, and non-coding transcripts longer than 200 nt were selected as lncRNAs. The differentially expressed lncRNAs were screened according to the screening criteria (log_2_ FC > 0.585 or < −0.585, *p* < 0.05), and analyzed through heat map.

### RNA-pull down

The Magnetic RNA–protein Pull-down Kit (Thermo) was used for lncRNA pull-down, according to the manufacturer’s instruction. In brief, SNHG12 or antisense RNA was labeled with biotin, streptavidin magnetic beads were added and incubated for 30 min at 24 °C with stirring. Subsequently, we washed unbound RNA with Tris and added RNA–protein binding buffer containing total protein to tubes containing streptavidin magnetic beads. After the magnetic beads were incubated with rotation at 4 °C for 90 min, they were washed with washing buffer and then incubated with elution buffer for 15 min at 37 °C with agitation. The supernatant was collected for silver staining.

### RNA immunoprecipitation (RIP)

RIP lysis buffer was used to lyse the cells. After cell lysis, 50 μL magnetic beads and 0.5 mL RIP wash buffer were added, vortexed briefly and mixed, and the supernatant was aspirated. Subsequently, 100 μL of RIP wash buffer was added to resuspend the magnetic beads and 5 μg of PBRM1/1 μg IgG antibody, and the beads were incubated at room temperature for 30 min. After brief centrifugation, the supernatant was removed and mixed with 0.5 mL RIP wash buffer. Then, 900 μL of RIP immunoprecipitation buffer was added to the magnetic beads-antibody mixture, and centrifuged at 4 °C at 14,000 rpm for 10 min. The supernatant was sucked into the magnetic bead antibody tube, incubated at 4 °C overnight for co-immunoprecipitation reaction, and 10 μL of the supernatant was sucked as “input.” The remaining portion of the magnetic beads was washed with RIP wash buffer, proteinase K buffer was added and incubated at 55 °C for 30 min before RNA extraction. qRT-PCR was performed for the detection of lncRNA.

### Chromatin immunoprecipitation (ChIP)

The ChIP assay kit (Millipore, New Bedford, MA) was used according to the manufacturer’s instructions. Proteins were cross-linked to DNA by fixing in formaldehyde. Anti-PBRM1 antibody was used to extract chromatin from the cells, and the chromatin-crosslinked DNA was sheared into 250–500 bp fragments. Putative PBRM1-binding fragments were detected using qRT-PCR.

### Statistical analysis

Statistical data are presented as the mean value ± standard deviation (SD). All data analyses in this study were performed using the GraphPad Prism 8.0 software. Student’s *t* test and analysis of variance were applied for comparison of differences between two groups or more than two groups, respectively. *P* value <0.05 was considered significant.

## Results

### Exosomes secreted by breast cancer cells promote the angiogenesis capacity of HUVECs

**Fig. 1:**
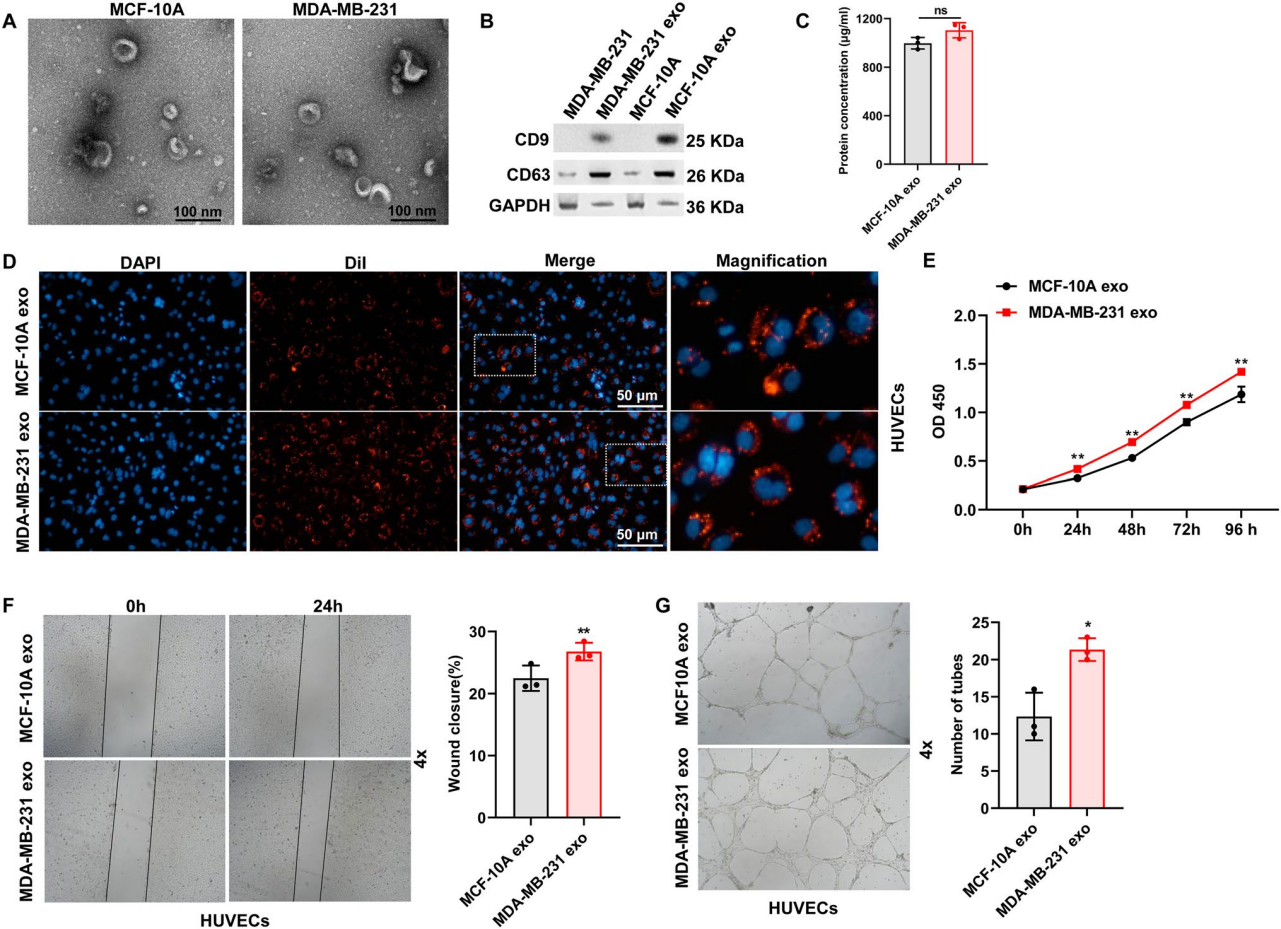
Breast cancer cell-derived exosomes enhanced HUVEC angiogenesis. Breast cancer cell-derived exosomes were isolated and characterized by transmission electron microscope (**A**) and western blot (**B**). Scale bar: 100 nm. **C** The protein concentration of exosomes was determined. **D** DiI-labeled exosomes were internalized by HUVECs. Scale bar = 50 μm. The role of breast cancer cell-derived exosomes in HUVECs proliferation (**E**), migration (**F**), and tube formation (**G**) was assayed by CCK-8, wound healing, and tube formation assay, respectively. MCF-10A exo represents MCF-10A cell-derived exosomes. MDA-MB-231 exo represents MDA-MB-231 cell-derived exosomes. Magnification: ×4. *n* = 3, * *p* < 0.05, ** *p* < 0.01

We first extracted exosomes from MDA-MB-231 cells and MCF-10A cells, and then characterized and identified them using TEM and western blot. The TEM analysis revealed that exosomes derived from both cell lines displayed a spherical-shaped appearance (Fig. 1A). Western blot analysis confirmed that exosomes from MCF-10A and MDA-MB-231 were enriched for the exosomal marker CD63 and CD9 (Fig. 1B). There was no significant difference in protein concentration between MCF-10A cell-derived exosomes and MDA-MB-231 cell-derived exosomes (Fig. 1C). Subsequently, DiI-labeled exosomes were co-cultured with HUVECs, demonstrating that HUVECs could internalize exosomes (Fig. 1D). HUVECs co-cultured with exosomes were used in a classical functional experiment to further investigate the biological functions of exosomes on HUVECs. First, CCK8 assays revealed that exosomes derived from MDA-MB-231 significantly enhanced the proliferation of HUVECs compared with that from MCF-10A (Fig. 1E). Additionally, wound healing and tube formation assays indicated that exosomes derived from MDA-MB-231 dramatically improved migration and tube formation capacities of HUVECs (Fig. 1F, G). These findings suggested that exosomes derived from breast cancer cells promoted angiogenesis of HUVECs.

### SNHG12 was upregulated in exosomes derived from breast cancer cells

**Fig. 2:**
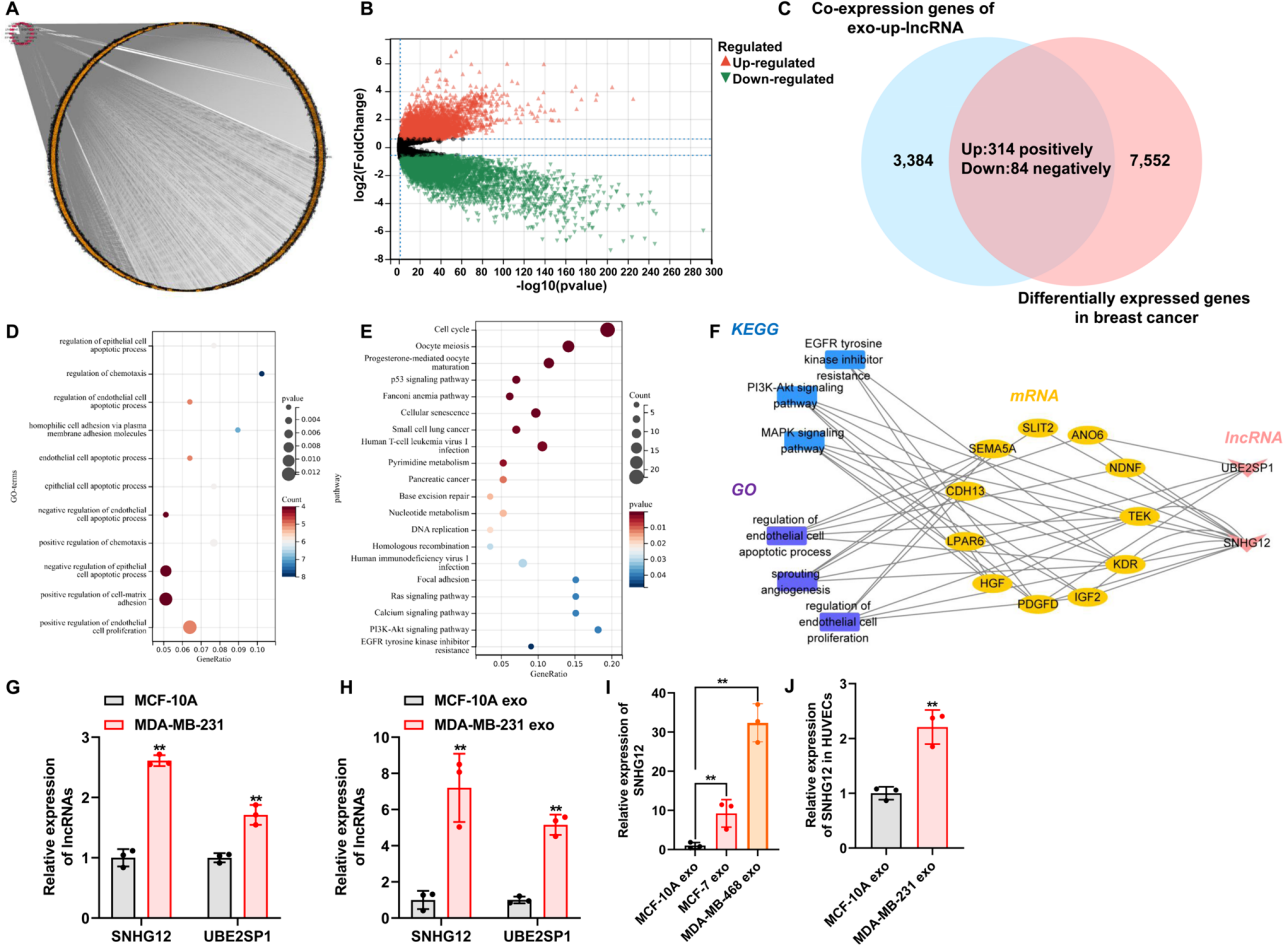
Screening and identification of lncRNAs associated with angiogenesis in exosomes. **A** Upregulated lncRNAs co-expressed genes in TCGA-Breast Cancer data. **B** The volcano plot showing differentially expressed genes between breast cancer and normal tissues using the TCGA database. **C** Venn diagram of co-expressed genes of exo-up-lncRNA and differentially expressed genes in breast cancer. GO (**D**) and KEGG (**E**) enrichment analysis of intersection genes. **F** The network diagram of lncRNAs-mRNAs-GO/KEGG. **G** The expression of lncRNA SNHG12 and UBE2SP1 was detected in breast cancer cells by qRT-PCR. **H** The expression of lncRNA SNHG12 and UBE2SP1 was detected in breast cancer cell-derived exosomes by qRT-PCR. **I** qRT-PCR was used to evaluate the expression of SNHG12 in exosomes from breast cancer cell lines MCF-7 and MDA-MB-468. **J** Breast cancer cell-derived exosomes increased SNHG12 levels in HUVECs. *n* = 3, ** *p* < 0.01

To identify the critical lncRNAs that regulate angiogenesis via exosomes, we screened exosomal lncRNAs based on the following criteria: (a) we first selected the exosomal upregulated lncRNAs (exo-up-lncRNAs) in the exosomes derived from MDA-MB-231 cells compared with those from MCF-10A cells, based on our previous sequencing results (Table S2) [17]; (b) the exo-up-lncRNAs were subjected to co-expression analysis to obtained their co-expressed genes using the TCGA-Breast Cancer database (Fig. 2A; Table S3); (c) we also analyzed differentially expressed genes between breast cancer and normal tissues using the TCGA database (Fig. 2B), then these differentially expressed genes were intersected with the above co-expressed genes of the exo-up-lncRNAs, resulting in 314 target genes that were upregulated in breast cancer and positively correlated with exo-up-lncRNAs, and 84 target genes that were downregulated and negatively correlated with exo-up-lncRNAs (Fig. 2C); (d) the above target genes of exo-up-lncRNAs were subjected to function analysis (Fig. 2D, E), and the lncRNAs that involved in the angiogenesis-associated GO or pathways were selected to establish a network of “lncRNAs—target genes—angiogenesis-associated GO or pathways” (Fig. 2F). Finally, SNHG12 and UBE2SP1 were selected as candidate lncRNAs. Next, we explored the expression of SNHG12 and UBE2SP1 in MDA-MB-231 cells and MCF-10A cells, and demonstrated that SNHG12 and UBE2SP1 were dramatically upregulated in MDA-MB-231 cells compared with MCF-10A cells (Fig. 2G). Meanwhile, the expression of SNHG12 and UBE2SP1 in exosomes derived from MDA-MB-231 cells was higher than that in exosomes derived from MCF-10A cells (Fig. 2H). Because SNHG12 was upregulated more than UBE2SP1, we selected SNHG12 to conduct the following experiments. To study the expression of SNHG12 in other breast cancer cell-derived exosomes, qRT-PCR was conducted to assess the expression of SNHG12 in exosomes from breast cancer cell lines MCF-7 and MDA-MB-468. The results showed that expression of SNHG12 was significantly higher in exosomes derived from MCF-7 and MDA-MB-468 cells than in exosomes derived from MCF-10A cells (Fig. 2I). Unsurprisingly, SNHG12 was significantly more abundant in HUVECs-treated with exosomes derived from MDA-MB-231 cells than in HUVECs co-cultured with MCF-10A cell-derived exosomes (Fig. 2J). These findings indicated that SNHG12 was an upregulated lncRNA derived from breast cancer cells and could be delivered by exosomes into HUVECs.

### Exosomes from breast cancer transport SNHG12 to promote HUVEC angiogenesis

**Fig. 3:**
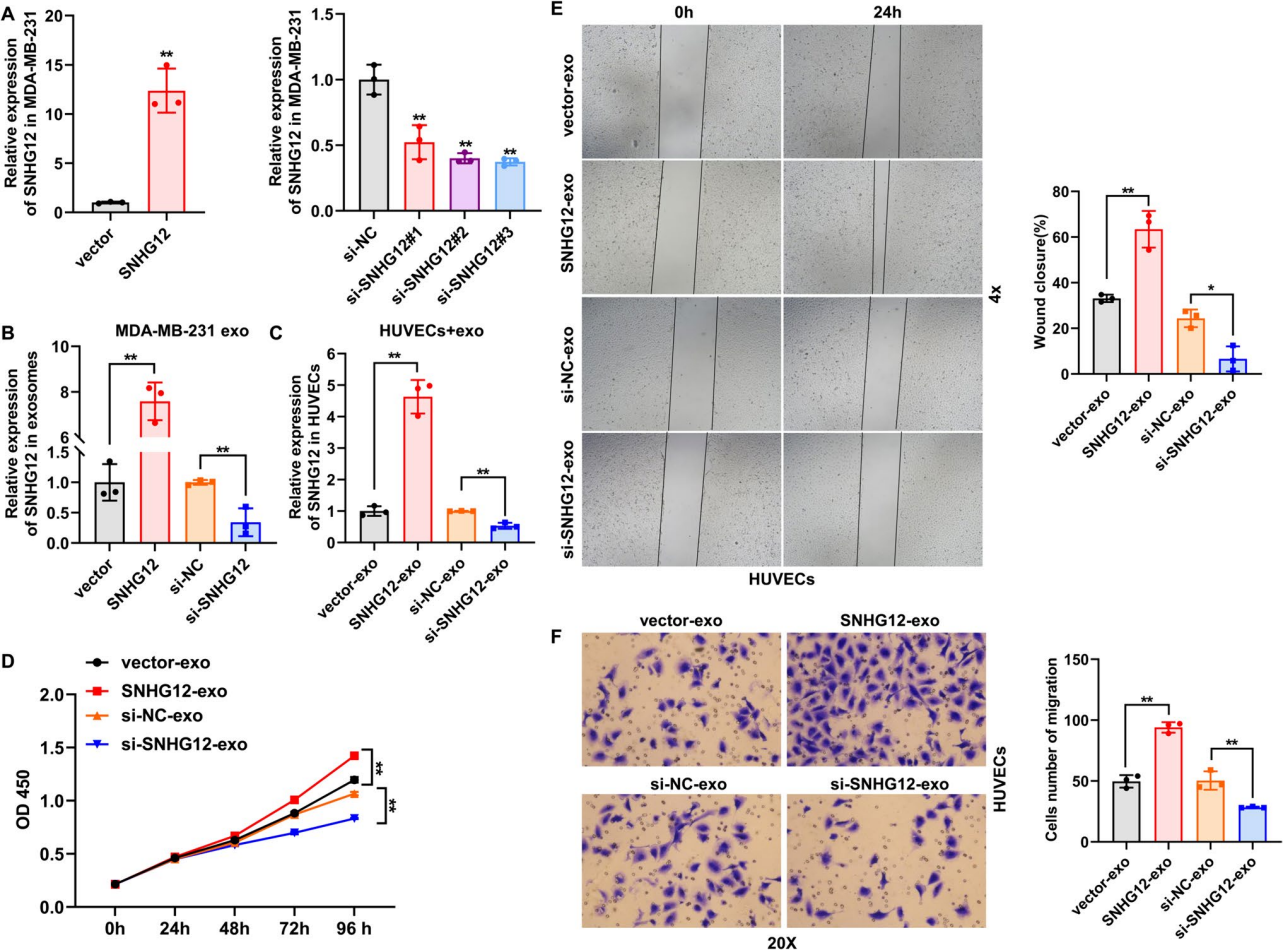
Exosomal SNHG12 enhanced HUVEC angiogenesis. **A** Transfection efficiency of SNHG12 overexpression and knockdown was determined by qRT-PCR. **B** qRT-PCR was used to detect the expression of SNHG12 in exosomes-overexpressed or deleted SNHG12. **C** qRT-PCR was used to detect the expression of SNHG12 in HUVECs co-cultured with exosomes-overexpressed or deleted SNHG12. **D** CCK-8 assay was performed to measure the effect of exosomal SNHG12 overexpression or silencing on HUVEC proliferation. **E** Wound healing assay was performed to determine the effect of exosomal SNHG12 overexpression or silencing on HUVEC migration. Magnification: ×4. **F** Transwell assay was performed to determine the effect of exosomal SNHG12 overexpression or silencing on HUVEC migration. Magnification: ×20. *n* = 3, * *p* < 0.05, ** *p* < 0.01

To investigate the role of exosomal SNHG12 on HUVECs, MDA-MB-231 cells were transfected with SNHG12 overexpression vector and si-SNHG12, and transfection efficiency was determined by qRT-PCR (Fig. 3A). Subsequently, we collected exosomes from MDA-MB-231 cells transfected with SNHG12 overexpression vector and siRNA fragment, respectively, and successfully obtained exosomes-overexpressed or deleted SNHG12 (Fig. 3B). Then, HUVECs were co-cultured with exosomes-deleted or overexpressed SNHG12. We observed that exosome-overexpressing SNHG12 efficiently increased SNHG12 levels in HUVECs (Fig. 3C). Conversely, exosome-deleted SNHG12 downregulated the expression of SNHG12 in HUVECs (Fig. 3C). Moreover, CCK8, wound healing, and transwell assays revealed that SNHG12 overexpression or knockdown in exosomes could promote or depress the proliferation and migration of HUVECs compared with the corresponding negative control groups (Fig. 3D–F). These findings suggest that exosomal SNHG12 can boost HUVEC angiogenesis.

### Exosomal SNHG12 strengthens angiogenesis and tumor growth in vivo

**Fig. 4:**
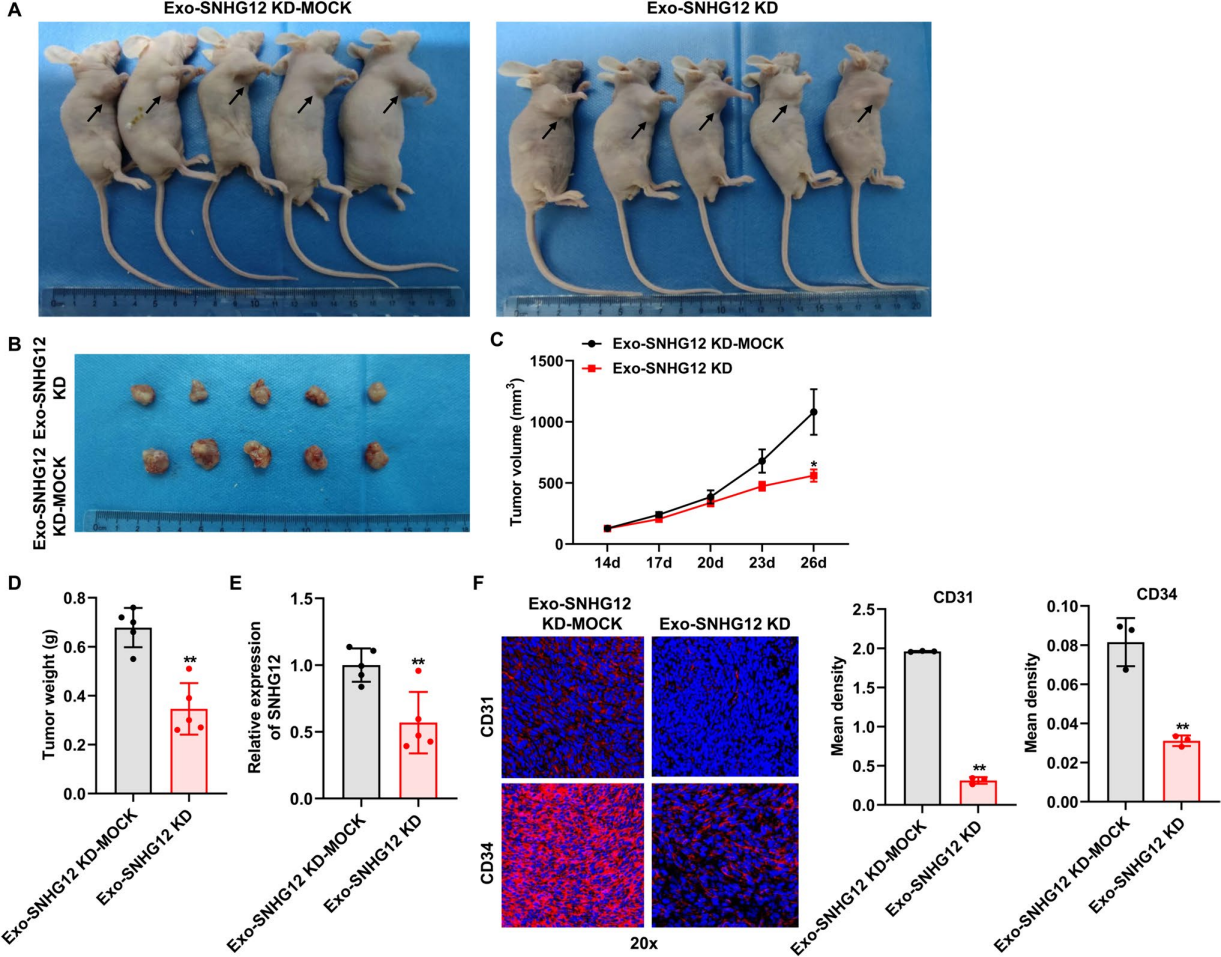
Exosomal with downregulated SNHG12 inhibited breast cancer tumorigenesis in vivo. **A**,**B** Representative pictures of tumors in each group. Exosomal with downregulated SNHG12 suppressed tumor volume (**C**) and weight (**D**), and decreased SNHG12 levels in tumor (**E**). *n* = 5. **F** Immunofluorescence was used to detect the expression of CD31 and CD34 in tumor tissue sections. Magnification: ×20. *n* = 3, * *p* < 0.05, ** *p* < 0.01

We further explored the possibility of exosome-delivered SNHG12 involved in HUVEC angiogenesis in vivo. We established a mouse subcutaneous tumor model by subcutaneous injection of MDA-MB-231 cells, and used exosome-deleted SNHG12 (named as Exo-SNHG12 KD) and corresponding negative control exosome (Exo-SNHG12 KD-MOCK) to treat the mice. As expected, Exo-SNHG12 KD could decelerate tumor growth compared with Exo-SNHG12 KD-MOCK (Fig. 4A, B). Consistently, the average tumor volume and weight were obviously reduced in the Exo-SNHG12 KD group compared with the Exo-SNHG12 KD-MOCK group (Fig. 4C, D). Meanwhile, SNHG12 knockdown in exosomes could significantly reduce the level of SNHG12 in tumor tissues (Fig. 4E). The blood vessels were detected in tumors via immunofluorescence using anti-CD31 and anti-CD34 antibodies. We found that the expression of endothelial markers CD34 and CD31 was observably decreased in the Exo-SNHG12 KD group compared with the Exo-SNHG12 KD-MOCK group, indicating a reduction in MVD (Fig. 4F). Collectively, these results demonstrated that exosomal SNHG12 had a promotive effect on angiogenesis and tumor growth.

### Screening of exosomal SNHG12 downstream target genes

**Fig. 5:**
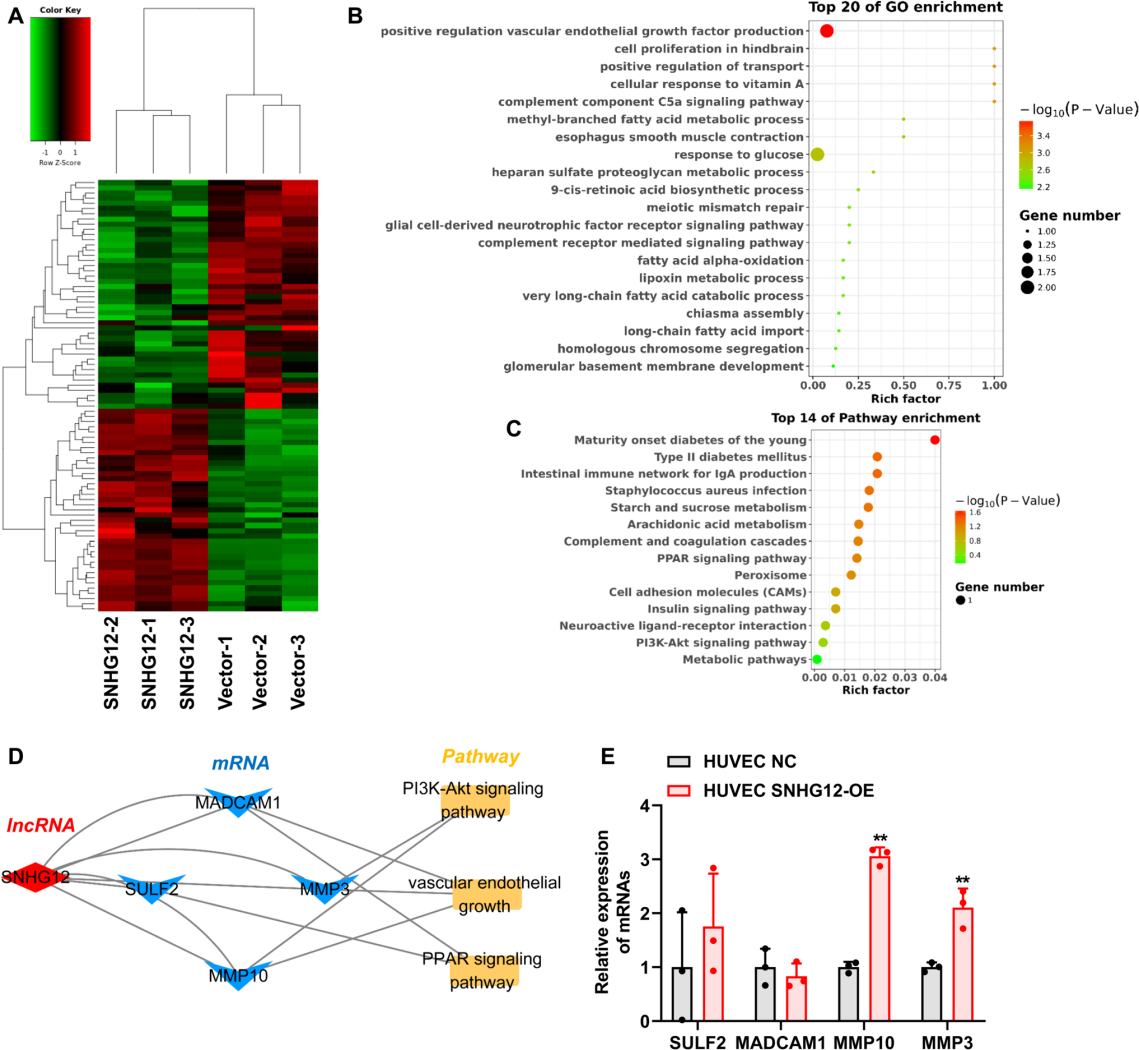
Screening of SNHG12 target genes. **A** Differentially expressed target genes of SNHG12 are shown in the heatmap. The differentially expressed SNHG12 target genes were subject to GO (**B**) and KEGG (**C**) analysis. **D** The network diagram of the SNHG12-mRNAs-pathway. **E** The selected target genes of SNHG12 were verified by qRT-PCR. *n* = 3, ** *p* < 0.01

To determine the potential target genes of SNHG12 in HUVECs, we performed RNA sequencing on HUVECs transfected with SNHG12 overexpression vectors. There were 39 upregulated mRNAs and 44 downregulated mRNAs identified in HUVECs after SNHG12 overexpression (Fig. 5A). Functional enrichment analysis showed that these differentially expressed genes were mainly enriched in positive regulation vascular endothelial growth factor production, cell proliferation in hindbrain, cell adhesion molecules (CAMs) and PI3K-Akt signaling pathway (Fig. 5B, C). MMP3, MMP10, SULF2, and MADCAM1 were selected as the candidate genes according to their biological functions that focused on angiogenesis, such as vascular endothelial growth, PI3K-Akt signaling pathway, and PPAR signaling pathway (Fig. 5D). qRT-PCR results confirmed that MMP10 and MMP3 were upregulated in HUVECs after SNHG12 overexpression, indicating they were potential targets gene of SNHG12 regulating HUVEC angiogenesis (Fig. 5E). Considering that MMP10 had the largest fold change, it was selected for subsequent validation.

### MMP10 has a pro-angiogenic effect on HUVECs

**Fig. 6:**
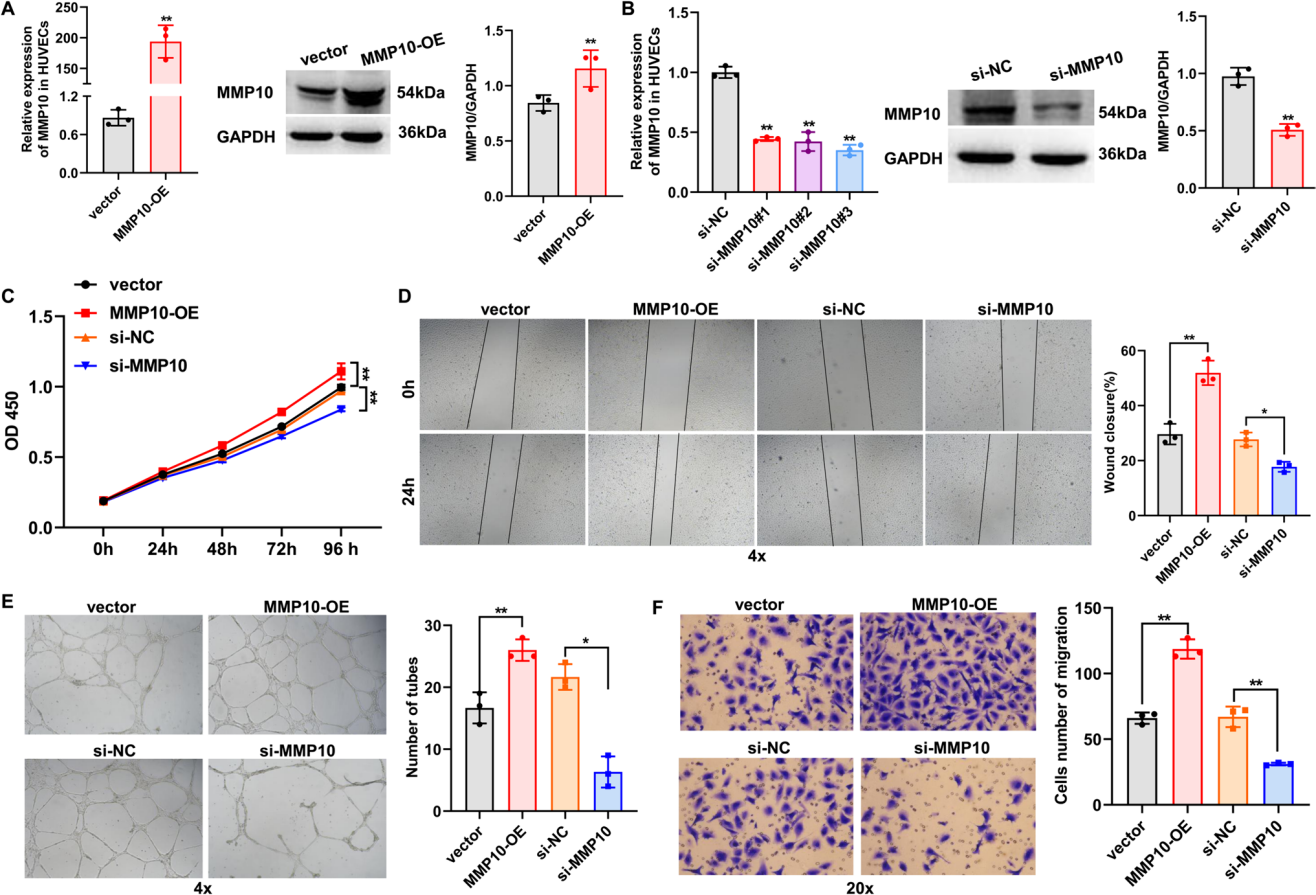
MMP10 enhanced HUVEC angiogenesis. **A**, **B** Transfection efficiency of MMP10 overexpression and knockdown was determined by qRT-PCR and western blot. **C** CCK-8 assay was conducted to determine the effect of MMP10 overexpression or silencing on HUVEC proliferation. **D** Wound healing assay was performed to determine the effects of MMP10 overexpression or silencing on HUVEC migration. Magnification: ×4. **E** Tube formation assay was performed to determine the effects of MMP10 overexpression or silencing on HUVEC tube formation. Magnification: ×4. **F** Transwell assay was performed to examine the effect of MMP10 overexpression or silencing on HUVEC migration. Magnification: ×20. *n* = 3, * *p* < 0.05, ** *p* < 0.01

To explore the potential function of MMP10 in HUVEC angiogenesis, the overexpression and interference efficiency of MMP10 in HUVECs was verified by qRT-PCR and western blot. The results indicated that the mRNA and protein expressions of MMP10 were remarkably increased or decreased in HUVECs after MMP10 overexpression or knockdown, respectively (Fig. 6A, B). Moreover, the upregulation or downregulation of MMP10 significantly intensified or suppressed the proliferation ability of HUVECs (Fig. 6C), as compared with their corresponding controls, respectively. Moreover, MMP10 overexpression prominently enhanced tube formation and migration capacities of HUVECs, whereas knockdown of MMP10 showed the opposite results in HUVECs (Fig. 6D–F). These findings suggest that MMP10 has a pro-angiogenic effect on HUVECs.

### SNHG12 directly interacts with PBRM1 to target MMP10

**Fig. 7:**
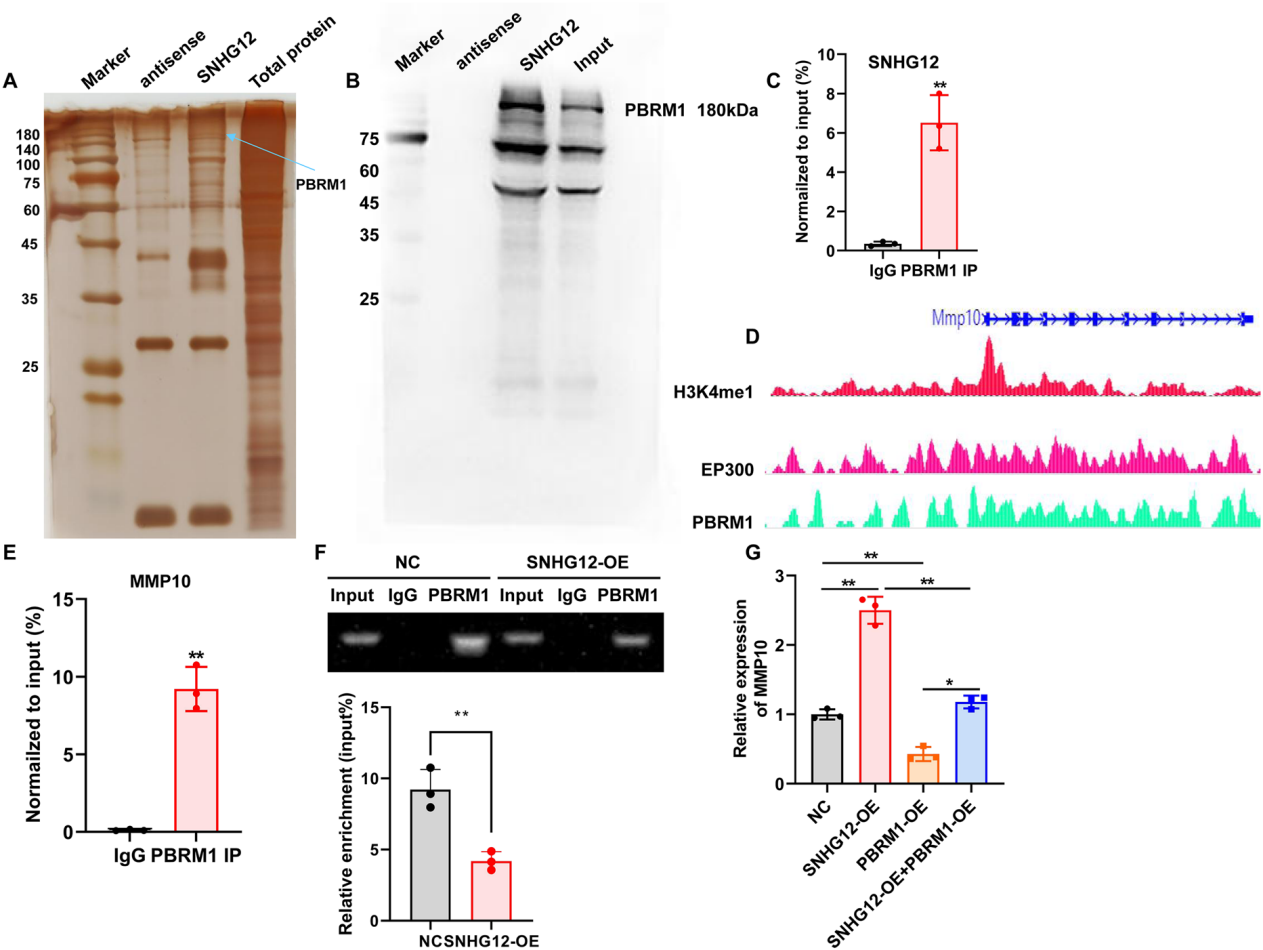
SNHG12 directly interacts with PBRM1 to upregulate MMP10. **A** Silver staining of SNHG12 pull down. **B** Western blot was used to detect SNHG12-binding proteins. **C** RIP-PCR was applied for the interaction between SNHG12 and PBRM1 in HUVECs. **D** The presumptive sites between PBRM1 and MMP10. **E** ChIP-PCR was applied for the interaction between MMP10 and PBRM1 in HUVECs. **F** SNHG12 overexpression weakens the interaction between MMP10 and PBRM1. **G** The MMP10 mRNA expression was verified by qRT-PCR. *n* = 3, * *p* < 0.05, ** *p* < 0.01

Accumulating evidence suggests that lncRNA-regulated target gene expression depends on lncRNA-protein interaction [20]. To explore the molecular mechanism by which SNHG12 regulates MMP10, we used the catRAPID database to predicted lncRNA-binding proteins and discovered that SNHG12 may bind to 100 proteins (Table S4). We further screened binding proteins that might regulate angiogenesis, and screened PBRM1 as a candidate binding protein of SNHG12, since its loss promotes angiogenesis [21]. We performed PBRM1 western blot on SNHG12 pull down products and found that SNHG12 could bind to PBRM1 (Fig.7A, B). Simultaneously, RIP-PCR revealed that PBRM1 directly interacted with SNHG12 (Fig. 7C). It has been reported that PBRM1 can bind to enhancer region of gene, resulting in inhibition of gene transcription [22], which prompts us to wonder whether PBRM1 also binds to MMP10 and inhibits the transcription of MMP10. Cistrome Data Browser predicted that PBRM1 could bind with the enhancer region of MMP10 promoter (Fig. 7D). To further verify the binding ability of PBRM1 to MMP10, we performed ChIP-PCR experiment. As revealed in Fig. 7E, PBRM1 directly bound to MMP10 mRNA. Furthermore, SNHG12 overexpression decreased the interaction between MMP10 and PBRM1 (Fig. 7F). Meanwhile, overexpression of SNHG12 enhanced MMP10 expression, which was partially reversed by PBRM1 overexpression (Fig. 7G). As a result, we confirmed that SNHG12 interacted directly with PBRM1 to upregulate MMP10 expression.

### Exosomal SNHG12 promotes HUVEC angiogenesis via MMP10 and PBRM1

**Fig. 8:**
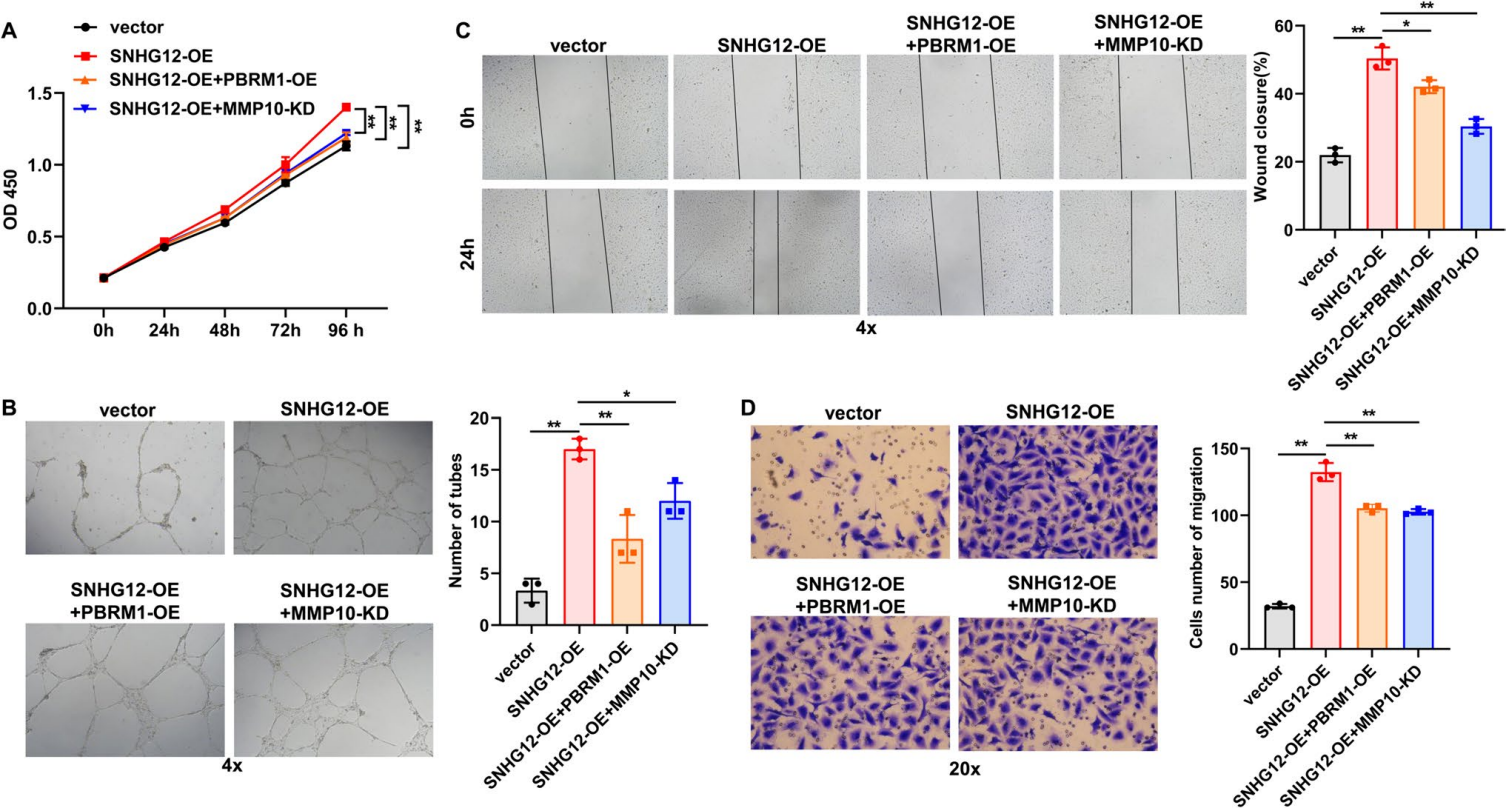
Exosomal SNHG12 facilitates HUVEC angiogenesis via MMP10 and PBRM1. **A** CCK-8 assay was used to evaluate the effect of MMP10 overexpression or PBRM1 silencing on exosomal SNHG12-mediated HUVEC proliferation. **B** Tube formation assay was performed to determine the effects of MMP10 overexpression or PBRM1 silencing on exosomal SNHG12-mediated HUVEC tube formation. Magnification: ×4. **C** Wound healing assay and **D** transwell assay were applied to determine the effects of MMP10 overexpression or PBRM1 silencing on exosomal SNHG12-mediated HUVEC migration. *n* = 3, * *p* < 0.05, ** *p* < 0.01

To investigate whether exosomal SNHG12 is involved in angiogenesis via MMP10 and PBRM1, we performed cell experiments. CCK8 assay, tube formation assay, wound healing assay and transwell assay demonstrated that SNHG12 overexpression in exosomes promoted proliferation, tube formation and migration of HUVECs (Fig. 8A–D). PBRM1 overexpression or MMP10 knockdown partially reversed the promotive effects of SNHG12 overexpression on HUVEC proliferation, tube formation, and migration (Fig. 8A–D). Collectively, these results suggest that exosomal SNHG12 can boost HUVEC angiogenesis via PBRM1 and MMP10.

## Discussion

Breast cancer is a malignant tumor in women, and angiogenesis is a critical process in the development of breast cancer [23]. The tumor cell microenvironment is critical for tumor angiogenesis, which is a key direction for tumor therapy [7]. Exosomes secreted by tumor cells regulate cancel cell proliferation, migration, and invasion in the tumor microenvironment via their cargo molecules [24]. Tao et al. demonstrated that exosomal EWSAT1 stimulated the sensitivity of vascular endothelial cells to induce osteosarcoma angiogenesis [25]. Ding et al. uncovered that exosomal lncRNA PART1 promoted esophageal cancer angiogenesis via the miR-302a-3p/CDC25A axis [26]. However, the function of exosomal lncRNAs on breast cancer angiogenesis remains unclear. In this study, exosomes derived from breast cancer cells promoted the angiogenesis of HUVECs, suggesting a regulatory role in angiogenesis. We demonstrated that SNHG12 was highly expressed in both breast cancer cells and their secreted exosomes, promoting HUVEC angiogenesis and tumor progression via PBRM1 and MMP10. Taken together, these findings suggest that breast cancer cell-derived exosomal SNHG12 was a key target for cancer therapy.

In the tumor microenvironment, exosomes are important bridges for endothelial cells and cancer cells to communicate with each other, mainly participating in cell process by regulating angiogenesis [27]. In this study, we demonstrated that breast cancer cell-derived exosomes promoted cell proliferation, tube formation and migration ability of HUVECs, but the relevant regulatory mechanism is still poorly understood. Emerging research has indicated that exosomes were responsible for cell-to-cell communication in the tumor microenvironment by delivering multiple molecules [28]. Therefore, it is necessary to identify the exosomal molecules that regulate angiogenesis.

As a new type of non-coding RNAs, lncRNAs participate in various physiological processes in organisms. For example, the metastasis of pancreatic cancer cells is tightly regulated by lncRNAs [29]. LncRNA-CDC6 was found to be highly expressed in breast cancer, causing tumor cells to proliferate and metastasize [30]. Using the TCGA database, we discovered that SNHG12 was highly expressed in MDA-MB-231 cells. Liu et al. reported that SNHG12 promotes the adverse progression of renal cell carcinoma cells, such as proliferation, migration, and invasion [31]. Similarly, SNHG12 overexpression exhibited a positive correlation with the progression of colon cancer [32]. These findings suggest that SNHG12 may be a key molecule in the malignant progression of breast cancer.

SNHG12 was highly expressed in breast cancer cell-derived exosomes and HUVECs co-cultured with exosomes, indicating that exosomal SNHG12 is an important molecule in angiogenesis. To confirm our hypothesis, HUVECs were co-cultured with exosome-overexpressing or -deleted SNHG12. We discovered that SNHG12 overexpression or knockdown in exosomes could facilitate or repress proliferation, angiogenesis and migration of HUVECs. Other studies have also reported that SNHG12 promotes angiogenesis during ischemic stroke [33]. In vivo studies further confirm our findings that SNHG12-deleted exosomes inhibited tumor progression and reduced MVD. MVD is considered a risk factor for metastasis and predicts poor prognosis in patients with breast cancer [34]. Previous studies have shown that overexpression of SNHG12 could enhance the recovery of neurological function and increase vascular density in the infarct border zone of middle cerebral artery occlusion mice [33]. Thus, these findings indicated that breast cancer cell-derived exosome delivered SNHG12 to promote HUVEC angiogenesis.

In addition, we identified MMP10 as a downstream target gene of SNHG12 in regulating angiogenesis in HUVECs. MMP10, a member of the matrix metalloproteinase (MMP) family, is involved in vascular development and lumen formation to promote cancer cell growth and migration [35]. In accordance with previous studies, MMP10 has pro-angiogenic effects [36, 37]. Moreover, we discovered the direct interaction between SNHG12 and PBRM1. PBRM1 deletion has been reported to be positively associated with angiogenesis [21]. Furthermore, PBRM1 has binding sites to the MMP10 promoter region, and PBRM1 has been reported to negatively regulate gene transcription [22]. Taken together, we hypothesized that SNHG12 directly binds to PBRM1, which prevents PBRM1 from binding to the enhancer region of the MMP10 promoter to upregulate MMP10, resulting in angiogenesis of HUVECs (Figure S1).

In conclusion, our findings demonstrated that exosomal SNHG12 regulates HUVEC angiogenesis to promote breast cancer progression by interacting with PBRM1 to target MMP10. Therefore, inhibition of exosomal SNHG12 has high application value in reducing the angiogenesis of HUVECs.

### Supplementary Information

Below is the link to the electronic supplementary material.

Figure S1. Proposed model underlying the roles of exosomal SNHG12-mediated PBRM1/MMP10 in angiogenesis. (TIF 2757 KB)

Supplementary file2 (XLSX 9 KB)

Supplementary file3 (XLSX 14 KB)

Supplementary file4 (XLSX 194 KB)

Supplementary file5 (XLSX 16 KB)

## Data Availability

The datasets supporting the conclusions of this article are available from the corresponding author upon reasonable request.
