# Supplementary figures and images for: Exosomal lncRNA SNHG12 promotes angiogenesis and breast cancer progression
Source: Breast Cancer. 2024 Jun 4;31(4):607–20. doi: 10.1007/s12282-024-01574-6 (PMC11194216; doi:10.1007/s12282-024-01574-6)

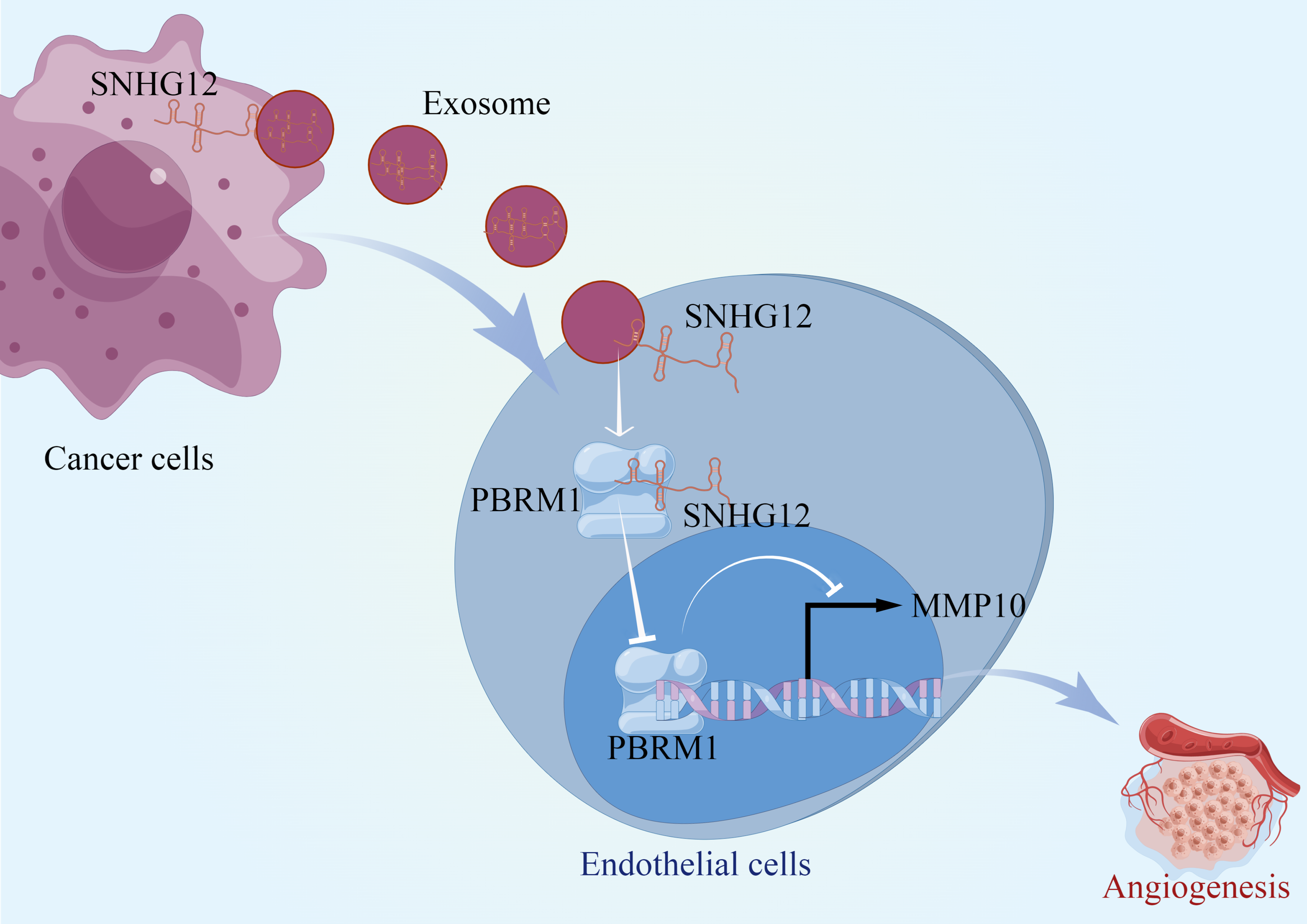

Supplement: Supplementary file 1 — Figure S1. Proposed model underlying the roles of exosomal SNHG12-mediated PBRM1/MMP10 in angiogenesis. (TIF 2757 KB) [file 12282_2024_1574_MOESM1_ESM.tif]
